# Biomechanical Effect of L_4_–L_5_ Intervertebral Disc Degeneration on the Lower Lumbar Spine: A Finite Element Study

**DOI:** 10.1111/os.12703

**Published:** 2020-05-31

**Authors:** Xin‐yi Cai, Meng‐si Sun, Yun‐peng Huang, Zi‐xuan Liu, Chun‐jie Liu, Cheng‐fei Du, Qiang Yang

**Affiliations:** ^1^ Tianjin Key Laboratory for Advanced Mechatronic System Design and Intelligent Control, School of Mechanical Engineering Tianjin University of Technology Tianjin China; ^2^ National Demonstration Center for Experimental Mechanical and Electrical Engineering Education Tianjin University of Technology Tianjin China; ^3^ Department of Spine Surgery The First Affiliated Hospital of Fujian Medical University Fuzhou Fujian China; ^4^ Department of Spine Surgery Tianjin Hospital Tianjin China

**Keywords:** Biomechanical effect, Disc degeneration, Finite element, Lower lumbar spine

## Abstract

**Objective:**

To ascertain the biomechanical effects of a degenerated L_4_–L_5_ segment on the lower lumbar spine through a comprehensive simulation of disc degeneration.

**Methods:**

A three‐dimensional nonlinear finite element model of a normal L_3_–S_1_ lumbar spine was constructed and validated. This normal model was then modified such that three degenerated models with different degrees of degeneration (mild, moderate, or severe) at the L_4_–L_5_ level were constructed. While experiencing a follower compressive load (500 N), hybrid moment loads were applied to all models to determine range of motion (ROM), intradiscal pressure (IDP), maximum von Mises stress in the annulus, maximum shear stress in the annulus, and facet joint force.

**Results:**

As the degree of disc degeneration increased, the ROM of the L_4_–L_5_ degenerated segment declined dramatically in all postures (flexion: 5.79°–1.91°; extension: 5.53°–2.62°; right lateral bending: 4.47°–1.46°; left lateral bending: 4.86°–1.61°; right axial rotation: 2.69°–0.74°; left axial rotation: 2.69°–0.74°), while the ROM in adjacent segments increased (1.88°–8.19°). The largest percent decrease in motion of the L_4_–L_5_ segment due to disc degeneration was in right axial rotation (75%), left axial rotation (69%), flexion (67%), right lateral bending (67%), left lateral bending right (67%), and extension (53%). The change in the trend of the IDP was the same as that of the ROM. Specifically, the IDP decreased (flexion: 0.592–0.09 MPa; extension: 0.678–0.334 MPa; right lateral bending: 0.498–0.205 MPa; left lateral bending: 0.523–0.272 MPa; right axial rotation: 0.535–0.246 MPa; left axial rotation: 0.53–0.266 MPa) in the L_4_–L_5_ segment, while the IDP in adjacent segments increased (0.511–0.789 MPa). The maximum von Mises stress and maximum shear stress of the annulus in whole lumbar spine segments increased (L_4_–L_5_ segment: 0.413–2.626 MPa and 0.412–2.783 MPa, respectively; adjacent segment of L_4_–L_5_: 0.356–1.493 MPa and 0.359–1.718 MPa, respectively) as degeneration of the disc progressively increased. There was no apparent regularity in facet joint force in the degenerated segment as the degree of disc degeneration increased. Nevertheless, facet joint forces in adjacent healthy segments increased as the degree of disc degeneration increased (extension: 49.7–295.3 N; lateral bending: 3.5–171.2 N; axial rotation: 140.2–258.8 N).

**Conclusion:**

Degenerated discs caused changes in the motion and loading pattern of the degenerated segments and adjacent normal segments. The abnormal load and motion in the degenerated models risked accelerating degeneration in the adjacent normal segments. In addition, accurate simulation of degenerated facet joints is essential for predicting changes in facet joint loads following disc degeneration.

## Introduction

Disc degeneration is a progressive condition that modifies the geometric morphology and biomechanical behavior of an intervertebral disc, eventually affecting its ability to transmit and distribute loads^1^. Lumbar disc degeneration is among the most common reasons for the development of lower back pain. Causes of disc degeneration are complex and multifactorial, caused by processes including aging, abnormal mechanical loads, and accidental damage^2^, ^3^, ^4^. It is known that the number of people experiencing lower back pain related to disc degeneration increases year on year^5^, ^6^. Degenerative disease of the lumbar spine is not only the cause of pain in numerous patients, it has become an excessive social burden in various countries^7^, ^8^, ^9^. Therefore, understanding the underlying mechanism that causes pain due to degeneration of lumbar discs is important when analyzing the basic principles of spine biomechanics, selection of optimal treatment options, or the development of new surgical devices.

Previous investigations have demonstrated that degeneration in the lumbar disc is often accompanied by ruptures, fissures, endplate sclerosis, formation of osteophytes, a reduction in disc height and nucleus pulposus volume, ligament failure, annulus fiber laxity, and changes in the material properties of the intervertebral disc^10^, ^11^, ^12^, ^13^, ^14^. In degenerated intervertebral discs, different morphological changes are present, individually or, more frequently, in various combinations. It is difficult to accurately simulate degenerative changes of the lumbar spine in finite element (FE) models by changing only a small number of intervertebral disc morphologies. A fundamental requirement for accurate simulation of disc degeneration in FE analysis is determining as far as possible the changes to intervertebral disc morphology and tissue properties.

Numerous *in vitro* experimental studies have analyzed the influence of disc degeneration on spinal biomechanics, including range of motion (ROM) of the lumbar spine^15^, ^16^, ^17^, facet contact force^18^, ^19^, intradiscal pressure (IDP)^13^, ^20^, and stress distribution in the vertebral body^21^. In particular, Kettler *et al*.^15^ explored stability in the early stages of lumbar disc degeneration and Mimura *et al*.^16^ studied the effect of disc degeneration on the multidirectional flexibility of the lumbar spine. These studies indicated that ROM in degenerated segments decreased during flexion–extension and lateral bending as disc degeneration progressed. There has also been progress in the study of the relationship between degenerative lumbar intervertebral discs and facet joint disease in the lumbar spine^19^. In addition, Adams *et al*. and McNally *et al*. investigated the mechanics of internal changes to intervertebral discs, finding a decrease in pressure when degenerated and analyzing the cause of that reduction in IDP^13^, ^20^. However, it is not sufficient to study the process of intervertebral disc degeneration using only *in vitro* experiments, because such tests cannot control the different degenerative changes, being highly dependent on the limited availability of cadavers. Likewise, the quality of cadaveric tissue can be problematic. FE analysis may, thus, provide an easier and more reliable method of investigation of mechanical changes due to intervertebral disc degeneration and has been extensively used in previous studies^22^, ^23^, ^24^, ^25^, ^26^. It has, to some extent, compensated for the shortcomings of *in vitro* experimental research.

Regarding FE studies of lumbar disc degeneration, Rohlmann *et al*.^22^ and Park *et al*.^23^ constructed three single‐segment lumbar models with different degrees of degeneration by changing both disc height and compressibility of the nucleus pulposus to study the influence of the degenerated disc on the biomechanical behavior of one motion segment. In addition, the second of these studies simulated degeneration by changing the material properties of the annulus fibrosus, ligaments, and nucleus pulposus^23^. Many single‐segment FE simulation studies have been conducted to investigate disc degeneration, but their common shortcoming is the inability to reflect the biomechanical changes in adjacent segments of the degenerated segment^12^, ^24^, ^25^, ^26^. Such biomechanical changes are also the focus of current studies. For example, Tang *et al*. simulated degeneration in the L_4_–L_5_ segment and developed three L_3_–L_5_ degenerated models and an anterior lumbar interbody fusion (ALIF) model of the L_3_–L_5_ segment. They compared the effects of ALIF and disc degeneration on the adjacent upper segmental disc and concluded that ALIF may promote adjacent upper segmental disc degeneration^27^. Ruberte *et al*. evaluated the effect of a single‐segment lumbar degenerated disc on the mechanical behavior of adjacent normal segments by changing the height of the intervertebral disc and the volume of the nucleus pulposus^28^. In addition, Park *et al*. simulated two degrees of disc degeneration and analyzed degeneration of intervertebral discs on intersegmental rotation, IDP, and facet contact forces on the whole lumbar spine. However, they only modified the material properties of the degenerated segment and disc height^29^. It is worth noting that there has also been some progress in the study of multi‐segmental simulation of lumbar disc degeneration^30^. Despite numerous studies of the mechanics of degenerated discs having been conducted by FE analysis, very few have comprehensively simulated lumbar disc degeneration.

Accordingly, the present study aimed to comprehensively simulate disc degeneration and evaluate the biomechanical effects of L_4_–L_5_ segmental intervertebral disc degeneration on the degenerated segment and adjacent normal segments in the lower lumbar spine.

## Materials and Methods

### *Development of Normal Lumbar Spine Model*

**Figure 1:**
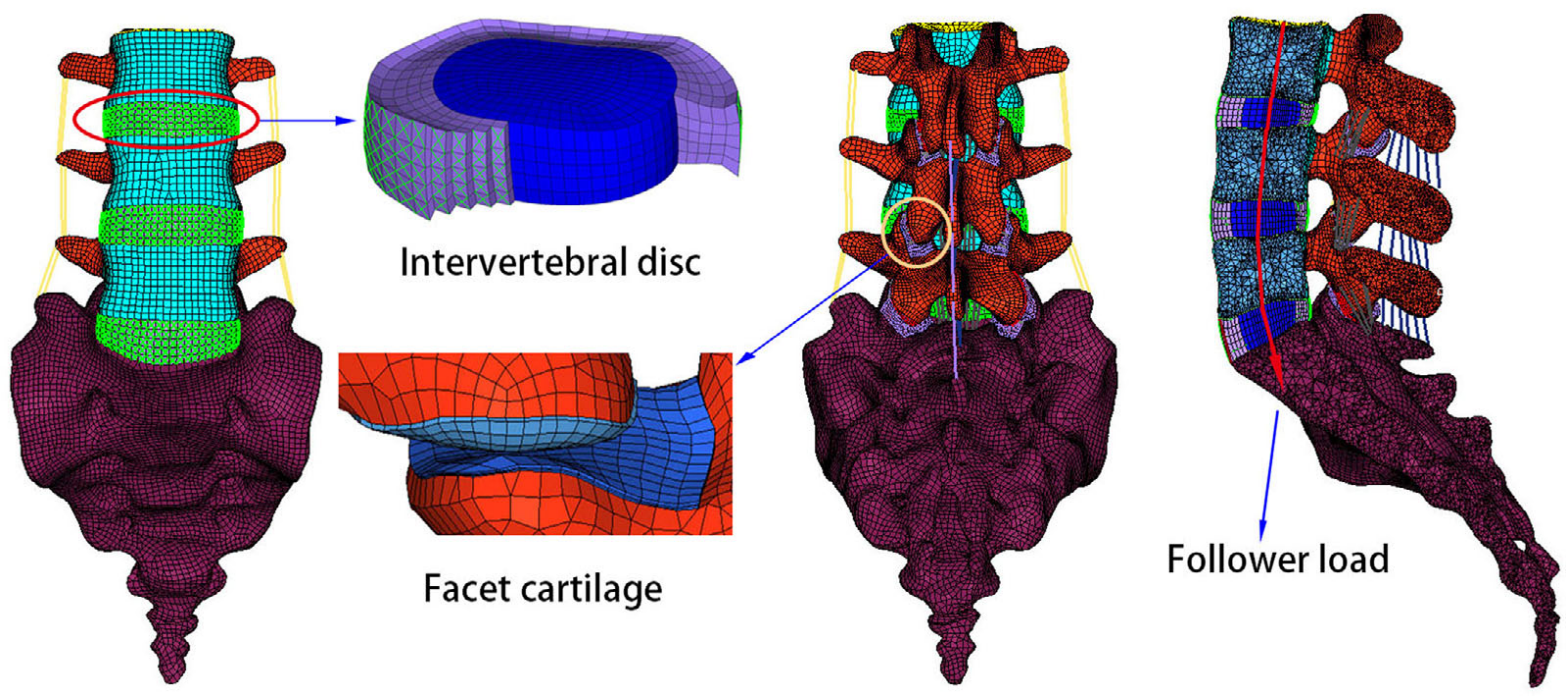
Three‐dimensional nonlinear finite element model of a normal lumbar spine (L_3_–S_1_).

The detailed steps for building a lumbar spine FE model were obtained from previous studies^31^, ^32^. Briefly, detailed geometric information of the lumbar spine (L_3_–S_1_) was derived from computed tomography (CT) images of a healthy male subject (age: 30 years, weight: 68 kg, height: 173 cm). According to the above geometric information of the lumbar spine, the normal lumbar spine FE model was developed. This normal FE model of the lumbar spine (L_3_–S_1_) is shown in Fig. 1 and includes posterior elements, cancellous bone, endplates, cortical bone, facet cartilage, intervertebral discs, and ligaments. The endplate in the model was composed of cartilage. The thickness of the cortical bone was set at 0.5 mm^33^. The mean initial gap between the facet cartilage surfaces was 0.1 mm and the interaction was defined as frictionless surface‐to‐surface contact. In addition, the facet cartilage had an inhomogeneous thickness, the distribution of thickness of which was derived from previously published literature^34^, ^35^. Intervertebral discs are composed of a nucleus pulposus, annulus ground, and fibers^36^, ^37^. The angle between fiber layers and the endplate surface was approximately ±30°^28^. The ligaments in the FE model included the anterior and posterior longitudinal ligaments, the interspinous ligament, the supraspinal ligament, the flaval ligament, the capsular ligament, and the intertransverse ligament. Of note, the fibers and ligaments were defined as tension‐only springs with nonlinear characteristics^38^. The types of element and material parameters of each component of the normal model are shown in Table 1 28, 30, 31, 32, 37, 38.

**Table 1:** Material properties and element types of the various spinal structures in the normal lumbar spine model^28^, ^30^, ^31^, ^32^, ^37^, ^38^

Component	Young's modulus (MPa)	Poisson's ratio	Element type	Element numbers
Cortical	12,000	0.3	C3D8R	1496
Cancellous	100	0.4	C3D4	83,619
Endplate	24	0.4	C3D8R	3168
Posterior element	3500	0.3	C3D4	155,673
Sacrum	5000	0.2	C3D4	200,295
Facet cartilage	Neo‐Hookean, C10 = 2		C3D8RH	4391
Annulus ground	Mooney–Rivlin, C1 = 0.18, C2 = 0.045		C3D8RH	3600
Nucleus pulposus	Mooney–Rivlin, C1 = 0.12, C2 = 0.03		C3D8RH	4320
Annulus fibers	Calibrated stress–strain curves		Spring	8640
Ligament	—		—	—
ALL	Calibrated deflection–force curves		Spring	15
PLL	Calibrated deflection–force curves		Spring	18
ISL	Calibrated deflection–force curves		Spring	18
SSL	Calibrated deflection–force curves		Spring	3
LF	Calibrated deflection–force curves		Spring	27
CL	Calibrated deflection–force curves		Spring	48
ITL	Calibrated deflection–force curves		Spring	12

ALL, anterior longitudinal ligament; CL, capsular ligament; FL, flaval ligament, ISL, interspinous ligament; ITL, intertransverse ligament; PLL, posterior longitudinal ligament; SSL, supraspinal ligament.

### *Development of Lumbar Disc Degeneration Models*

**Figure 2:**
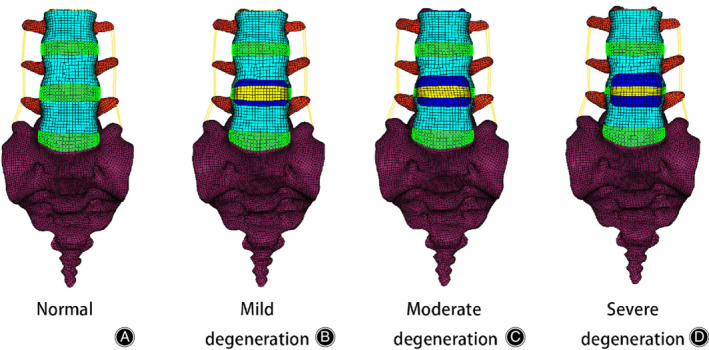
Anterior views of a normal and three degenerative three‐dimensional nonlinear finite element models of the lumbar spine (L_3_–S_1_). (A) Normal model: material properties (no degeneration), normal disc height (no degeneration), no anterior osteophytes. (B) Mild degeneration: material properties (mild degeneration), 20% reduction in disc height, anterior osteophyte size representing 10%. (C) Moderate degeneration: material properties (moderate degeneration), 40% reduction in disc height, anterior osteophyte size representing 20%. (D) Severe degeneration: material properties (severe degeneration), 60% reduction in disc height, anterior osteophytes size representing 30%.

**Figure 3:**
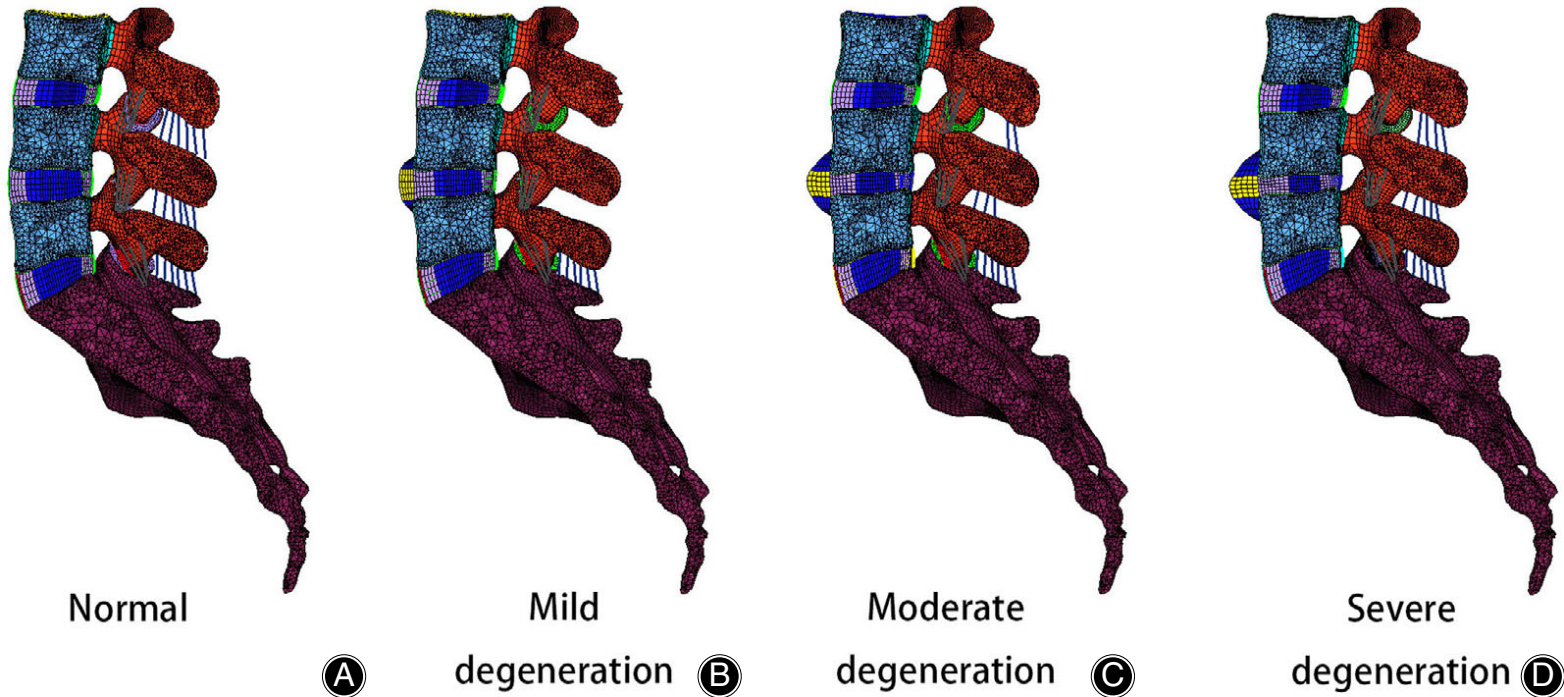
Sagittal views of a normal and three degenerative three‐dimensional nonlinear finite element models of the lumbar spine (L_3_–S_1_). (A) Normal model: the material properties (no degeneration), normal disc height (no degeneration), no anterior osteophytes. (B) Mild degeneration: material properties (mild degeneration), 20% reduction in disc height, anterior osteophyte size representing 10%. (C) Moderate degeneration: material properties (moderate degeneration), 40% reduction in disc height, anterior osteophyte size representing 20%. (D) Severe degeneration: material properties (severe degeneration), 60% reduction in disc height, anterior osteophytes size representing 30%.

In the present study, three variables of intervertebral disc morphology (disc height, formation of anterior osteophytes, and area of nucleus pulposus) were modified to construct three lumbar spine FE models simulating mild, moderate, and severe degeneration at the L_4_–L_5_ level (Figs 2 and 3). The L_4_–L_5_ segment was selected to simulate disc degeneration due to a higher incidence of degeneration^28^, ^39^, ^40^.

**Figure 4:**
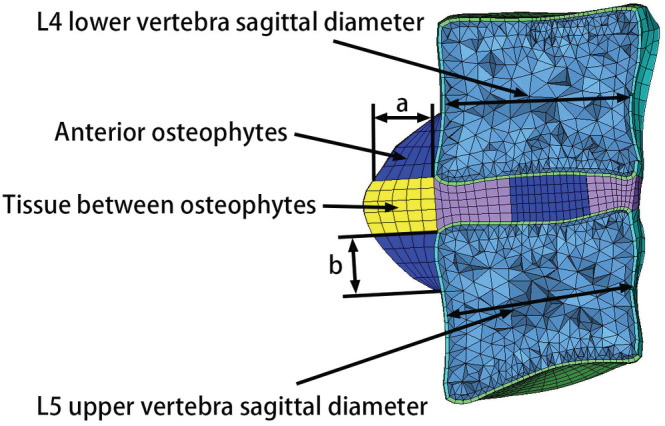
Schematic diagram of the vertebral sagittal diameter and anterior osteophyte dimensions.

The specific values and proportion of three variables of intervertebral disc morphology are shown in Table 2 12, 22, 24, 28, 42. Compared with a normal intervertebral disc, disc height in mild, moderate, and severe degeneration decreased by 20%, 40%, and 60%, respectively^22^. The values of height and length of osteophytes in the sagittal plane were equal (Fig. 4). Osteophytes in the L_4_ lower vertebra were classified as 10% (mild degeneration), 20% (moderate degeneration), or 30% (severe degeneration) of the normal sagittal diameter of an L_4_ vertebra^12^, ^24^. Classification of osteophytes of the L_5_ upper vertebra was the same as described above. As the degree of degeneration increased, the volume of the nucleus pulposus gradually decreased^41^, ^42^. To maintain a constant intervertebral disc volume, decreased nucleus pulposus volume was replaced with elements representing annulus ground. Changes in the nucleus pulposus and the annulus cross‐sectional area are shown in Table 2. In addition, the material properties of the endplate^12^, ^24^, ^43^, ^44^, ^45^ and intervertebral disc^12^, ^24^, ^28^ in the degenerated segment were also modified to further simulate disc degeneration (Tables 3 and 4). Modification of the endplate material properties simulated endplate sclerosis. In addition, analysis using frozen sections has indicated that the material properties of osteophytes and the soft tissue between them are similar to those of cancellous bone and annulus ground, respectively^12^, ^43^, as presented in Table 4 12, 43, 44.

**Table 2:** Changes in three variables of lumbar intervertebral disc geometry morphology^12^, ^22^, ^24^, ^28^, ^42^

Variables of lumbar disc morphology	Normal	Mild	Moderate	Severe
**Height loss**				
Anterior disc height loss (mm)	0	2.42 (20%)	4.84 (40%)	7.26 (60%)
Posterior disc height loss (mm)	0	1.55 (20%)	3.50 (40%)	5.25 (60%)
**Anterior osteophytes**				
Osteophytes of L_4_ low vertebral (mm)	0	3.01 (10%)	6.02 (20%)	9.03 (30%)
Osteophytes of L_5_ upper vertebral (mm)	0	2.96 (10%)	5.92 (20%)	8.88 (30%)
**The changes of intervertebral disc area**				
Nucleus pulposus area (mm^2^)	613	463	333	223
Annulus ground area (mm^2^)	762	912	1042	1152

Height loss: Compared with a normal intervertebral disc, disc height in mild, moderate, and severe degeneration decreased by 20%, 40%, and 60%, respectively. The percentage of anterior disc height loss and posterior disc height loss are the same. For example, the 20% of anterior disc height loss of normal intervertebral disc is 2.42 mm

Anterior osteophytes: Osteophytes in the L_4_ lower vertebra were classified as 10% (mild degeneration), 20% (moderate degeneration), or 30% (severe degeneration) of the normal sagittal diameter of an L_4_ vertebra. Classification of osteophytes of the L_5_ upper vertebra was the same as described above. For example, 10% of the normal sagittal diameter of an L_4_ vertebra is 3.01 mm.

**Table 3:** Material properties and element types of the annulus ground and nucleus pulposus in all lumbar spine models^12^, ^24^, ^28^

The degree of lumbar disc degeneration	Hype‐elastic Mooney–Rivlin		Element type
	C1	C2	
**No degeneration**			
Annulus ground	0.18	0.045	C3D8RH
Nucleus pulposus	0.12	0.03	C3D8RH
**Mild degeneration**			
Annulus ground	0.4	0.1	C3D8RH
Nucleus pulposus	0.14	0.035	C3D8RH
**Moderate degeneration**			
Annulus ground	0.6	0.15	C3D8RH
Nucleus pulposus	0.17	0.041	C3D8RH
**Severe degeneration**			
Annulus ground	0.9	0.23	C3D8RH
Nucleus pulposus	0.19	0.045	C3D8RH

**Table 4:** Material properties and element types of the endplate, osteophytes and tissue between osteophytes in all lumbar spine models^12^, ^24^, ^43^, ^44^, ^46^

Degree of lumbar disc degeneration	Young's modulus (MPa)	Poisson's ratio	Element type
**No degeneration**			
Endplate	24	0.4	C3D8R
Osteophytes	—	—	—
Tissue between osteophytes	—	—	—
**Mild degeneration**			
Endplate	24	0.4	C3D8R
Osteophytes	100	0.4	C3D8R
Tissue between osteophytes	Mooney–Rivlin, C1 = 0.4, C2 = 0.1		C3D8RH
**Moderate degeneration**			
Endplate	50	0.4	C3D8R
Osteophytes	100	0.4	C3D8R
Tissue between osteophytes	Mooney–Rivlin, C1 = 0.6, C2 = 0.15		C3D8RH
**Severe degeneration**			
Endplate	100	0.4	C3D8R
Osteophytes	100	0.4	C3D8R
Tissue between osteophytes	Mooney–Rivlin, C1 = 0.9, C2 = 0.23		C3D8RH

### *Loading and Boundary Conditions*

In the present study, a hybrid moment load was used to compare differences between the healthy and the three degenerated models, because such a load required the same end conditions of these lumbar spine FE models. That is, in each posture, the L_3_–S_1_ total ROM of each degenerated model was the same as for the normal model. The resultant moment loading required to achieve the same L_3_–S_1_ rotational motion as the normal model is listed in Table 5. During loading, the sacrum was fixed in six possible types of motion. When placed under a follower load (500 N), the moment loading described above was applied to the superior surface of the L_3_ endplate in all models. This method of applying a follower load is the same as described in the published literature^29^, ^47^.

**Table 5:** Moment loading applied to each finite element model of the lumbar spine (Nm)

Types	Flexion	Extension	RB	LB	RAR	LAR
No degeneration	7.5	7.5	7.5	7.5	7.5	7.5
Mild degeneration	11.3	10.6	11	11	11.9	11.7
Moderate degeneration	11.9	11.7	11.5	11.6	12.3	12.1
Severe degeneration	12.3	13.1	12.6	12.7	12.8	12.9

Dashes refer to the normal lumbar spine model lack osteophytes and tissue between osteophytes. LAR, left axial rotation; LB, left bending; RAR, right axial rotation; RB, right bending.

### *Calibration and Validation*

The process of calibration was conducted by changing correction factors for the collagen fibers and ligaments, in accordance with previously published literature^48^, ^49^. The detailed process of calibration was described elsewhere^31^, ^32^. The ROM of the lumbar spine in each segment when placed under a pure moment load (flexion, 8 Nm; extension, 6 Nm; lateral bending, 6 Nm; axial rotation, 4 Nm) and disc compression under a follower compressive load of 1200 N were calculated. The motion of each segment of the normal model was compared with previous cadaveric specimen data to validate the normal model^50^. Boundary and loading conditions were set and replicated *in vitro*. All simulation work was conducted using commercial FE software (Abaqus 6.11; Dassault Systemes Simulia, Pennsylvania, USA).

## Results

### *Calibration and Validation*

**Figure 5:**
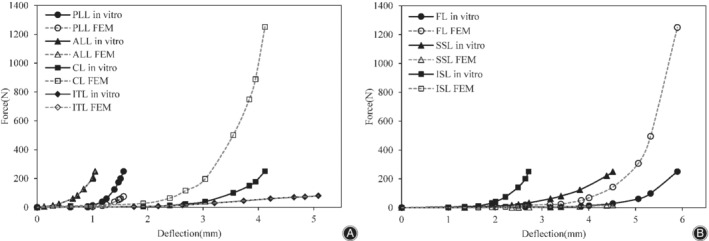
Calibration results of seven major ligaments of the lumbar spine finite element model: (A) anterior longitudinal ligament (ALL), capsular ligament (CL), intertransverse ligament (ITL), and posterior longitudinal ligament (PLL); and (B) flaval ligament (FL), interspinous ligament (ISL), and supraspinal ligament (SSL).

**Figure 6:**
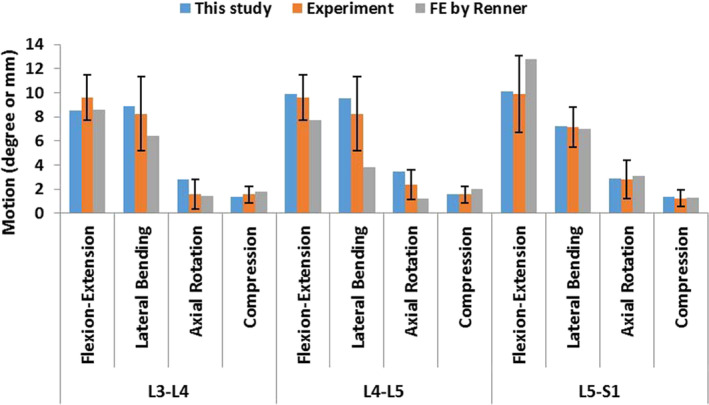
Comparison of finite predicted data of the normal model (range of motion and disc compression of each segment) and experimental data of clinical specimens by Renner *et al*.^50^.

The calibration results are presented in Fig. 5A,B and the validation results are presented in Fig. 6. The optimized calibration factors of fibers and ligaments were as follows: fibers, 0.49; ALL, 1.0; PLL, 0.3; ITL, 1.0; FL, 5.0; SSL, 0.07; ISL, 0.08, and CL, 5.0. Figure 6 displays predicted data from FE analysis of the normal model (ROM and disc compression of each segment) compared with experimental data of specimens by Renner *et al*.^50^. The ROM and disc compression of each segment in each posture closely matched the *in vitro* experimental data. Therefore, the model was considered to be calibrated and validated, and was used to further study the biomechanical response of the lumbar spine under disc degeneration.

### *Range of Motion*

**Figure 7:**
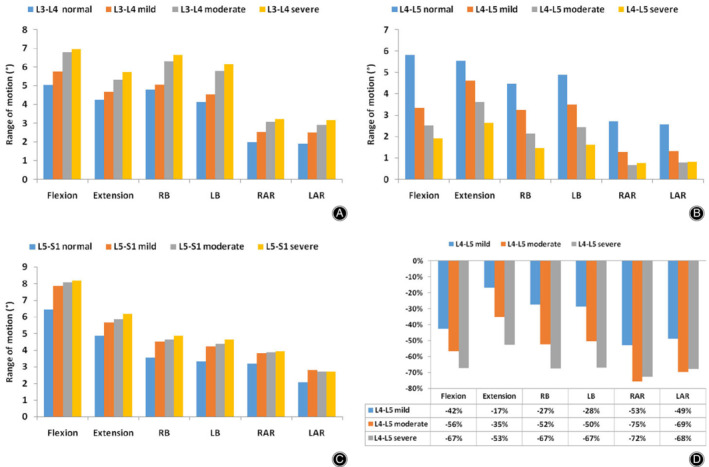
Range of motion (ROM) in normal and degenerated lumbar spine finite element (FE) models in six directions (LAR, left axial rotation; LB, left bending; RAR, right axial rotation; RB, right bending). (A) ROM of the L_3_–L_4_ segment in all lumbar spine FE models. (B) ROM of the L_4_–L_5_ segment in all lumbar spine FE models. (C) ROM of the L_5_–S_1_ segment in all lumbar spine FE models. (D) Percentage change in ROM of the L_4_–L_5_ degenerated segment with respect to L_4_–L_5_ segments of the normal model.

The ROM of the L_4_–L_5_ degenerated segment and adjacent normal segments in six directions under hybrid moment loading are shown in Fig. 7A–C. In a degenerated L_4_–L_5_ segment, the ROM gradually decreased with increasing disc degeneration. In a normal segment adjacent to an L_4_–L_5_ degenerated segment, the ROM gradually increased as the degree of disc degeneration increased. The percentage change in the ROM in the L_3_–L_4_ segment is larger than that of the L_5_–S_1_ segment. The percentage change in the ROM of the L_4_–L_5_ degenerated segment relative to the L_4_–L_5_ segment of the normal lumbar spine model is shown in Fig. 7D.

### *Intradiscal Pressure*

**Figure 8:**
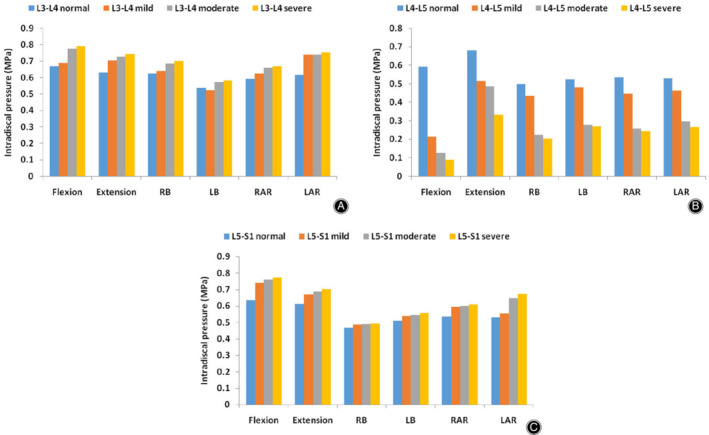
Intradiscal pressure (IDP) of the normal and degenerated lumbar spine finite element (FE) models in six directions. (A) IDP of the L_3_–L_4_ segment in all lumbar spine FE models. (B) IDP of the L_4_–L_5_ segment in all lumbar spine FE models. (C) IDP of the L_5_–S_1_ segment in all lumbar spine FE models.

In different postures, the IDP of the L_4_–L_5_ degenerative segment and adjacent normal segments when placed under hybrid moment loading are shown in Fig. 8A–C. Compared with the normal model, the IDP of the L_4_–L_5_ degenerated segment declined dramatically in all postures as the degree of disc degeneration increased. In contrast, the IDP in the adjacent normal segments of the L_4_–L_5_ degenerated segments increased with increasing disc degeneration.

### *Maximum Von Mises Stress in the Annulus*

**Figure 9:**
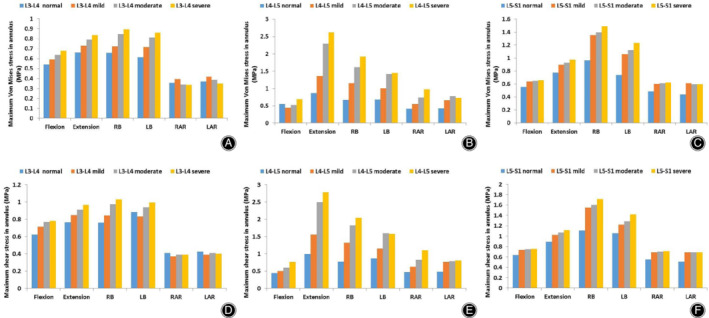
Maximum von Mises and shear stress in the annulus of the normal and degenerated lumbar spine finite element (FE) models in six directions. (A) Maximum von Mises stress in the annulus of the L_3_–L_4_ segment in all lumbar spine FE models. (B) Maximum von Mises stress in the annulus of the L_4_–L_5_ segment in all lumbar spine FE models. (C) Maximum von Mises stress in the annulus of the L_5_–S_1_ segment in all lumbar spine FE models. (D) Maximum shear stress in the annulus of the L_3_–L_4_ segment in all lumbar spine FE models. (E) Maximum shear stress in the annulus of the L_4_–L_5_ segment in all lumbar spine FE models. (F) Maximum shear stress in the annulus of the L_5_–S_1_ segment in all lumbar spine FE models.

The maximum von Mises stress in the annulus of the L_3_–S_1_ lumbar segment in six different postures under varying hybrid moment loading are shown in Fig. 9A–C. Under such hybrid moment loading, the maximum von Mises stress in the annulus of the L_4_–L_5_ degenerated segment increased with increasing degree of degeneration. Compared with a normal lumbar spine model, increasing degeneration of the disc resulted in an increase in maximum von Mises stress in the annulus of the adjacent normal segments, with the exception of the L_3_–L_4_ segment during axial rotation.

### *Maximum Shear Stress in the Annulus*

Maximum shear stress in the annulus of all lumbar FE models in six directions of loading under hybrid moment loading is shown in Fig. 9D–F. Maximum shear stress in the annulus of the L_4_–L_5_ degenerated segment increased with increasing disc degeneration. In the segments adjacent to the L_4_–L_5_ degenerated segment, in addition to axial rotation of the L_3_–L_4_ segments, the maximum shear stress in the annulus of the adjacent normal segments increased as the degree of degeneration increased.

### *Facet Joint Force*

**Figure 10:**
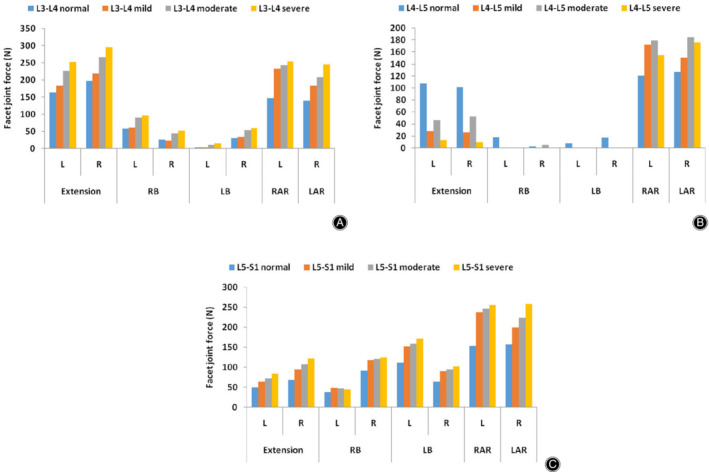
Facet joint force in normal and degenerated lumbar spine finite element (FE) models in six directions (L, left; R, Right). (A) Facet joint force of the L_3_–L_4_ segment in all lumbar spine FE models. (B) Facet joint force of the L_5_–S_1_ segment in all lumbar spine FE models. (C) Facet joint force of the L_4_‐L_5_ segment in all lumbar spine FE models.

The gap between facet cartilage during flexion was so large that the facet joint force was very small or even zero, so facet joint force is not shown in flexion. In the present study, facet joint forces on the left and right sides of the lumbar spine were recorded. The predicted facet joint forces in different lumbar FE models in different postures under loading using a hybrid moment are shown in Fig. 10A–C. In the L_4_–L_5_ degenerated segment, compared with the normal lumbar spine model, facet joint forces decreased during extension and increased in axial rotation. In lateral bending, the facet joint force was small and irregular. In summary, the change in facet joint force is irregular in an L_4_–L_5_ degenerated segment. However, facet joint forces on the segments adjacent to the L_4_–L_5_ degenerated segment gradually increased as the extent of disc degeneration increased.

## Discussion

A three‐dimensional nonlinear normal lumbar spine FE model of the L_3_–S_1_ segments was constructed, then validated by comparing data with previously published experimental data. Three different degrees of lumbar disc degeneration (mild, moderate, and severe) were modeled by modifying both the geometry of the intervertebral disc and the associated material properties. Using these models, we evaluated the effect of intervertebral disc degeneration in the L_4_–L_5_ segment on the lower lumbar spine.

It is common to use a hybrid moment loading to simulate various postures in lumbar spine FE studies, especially when studying lumbar spine degeneration^27^, ^28^, ^29^, ^32^. When the lumbar spine is degenerated, the ROM of the degenerated segment tends to decrease, but the patient often needs to maintain the total ROM originally present. During this time, the lumbar spine of the patient may cause pain. The key aim of using hybrid moment loading is to maintain the ROM of the degenerated lumbar spine models at the same value as the total ROM of the normal lumbar spine model. That is, we simulated the physiological state of the lumbar spine after the patient returned to the normal ROM and further| explored the mechanisms underlying pain in disc degeneration.

Progressive disc degeneration affects the pattern of motion of the diseased disc and its adjacent discs. In the present study, with increasing degree of disc degeneration, the results indicated that the ROM of the L_4_–L_5_ degenerated segment decreased dramatically and that of its healthy adjacent segments increased in all postures. Because the total ROM (L_3_–S_1_) of the healthy model was the same as that of the degenerated models, the adjacent intervertebral disc levels compensated for changes in motion of the degenerated disc. Several previous studies have reached the same conclusion for the trend of segmental rotation predicted by the finite element model in the present study^23^, ^27^, ^28^, ^29^, ^30^. However, a number of *in vitro* experimental^15^, ^16^, ^51^, ^52^ and finite element studies^22^ have indicated that the ROM of the degenerated segments increases in axial rotation. That may be because degenerated discs are often accompanied by fissures and ruptures during *in vitro* experimental studies^10^. Such fissures and ruptures reduce the limit of the ROM of discs in axial rotation. In addition, the reason for the difference, as shown by the research results of Rohlmann *et al*.^22^, may be due to different methods of modeling. Rohlmann *et al*.^22^ only changed one geometrical variable, disc height, to simulate disc degeneration. In comparison, we simulated the formation of anterior osteophytes and the reduction in nucleus pulposus volume. The reduced nucleus pulposus was replaced by annulus ground material, which had greater stiffness. In addition, anterior osteophytes limited the motion of the degenerated disc.

With progressive disc degeneration, the volume of the nucleus pulposus decreased, as did the ability of the tissue to stay hydrated, leading to further loss in disc height and changes in the tissue properties of the disc^3^, ^13^, ^41^. Due to the reasons above, transfer in load transmission from the nucleus pulposus to the annulus was evident with advancing disc degeneration^28^. The above phenomena are clearly evident in the present study. With increasing numbers of disc degenerative pathologies, the IDP of the L_4_–L_5_ degenerative segments dramatically declined in six postures, while maximum von Mises stress and shear stress in the annulus of the L_4_–L_5_ degenerated segment increased with an increasing degree of degeneration. These changes are in agreement with previous results, as reported in the literature^22^, ^23^, ^28^, ^29^. In addition, the maximum von Mises stress and the maximum shear stress in the annulus and the IDP in normal segments adjacent to the L_4_–L_5_ degenerated segment increased as the degree of disc degeneration increased. A number of studies support the conclusions above^28^, ^29^. From an anatomical and physiological point of view, as the load‐supporting function is progressively transferred from the nucleus pulposus to the annulus, intervertebral disc degeneration is initiated^53^, ^54^. In summary, degenerated discs change the capability of the degenerated segment and its adjacent normal segments to transmit and distribute loads.

Facet joint force is very sensitive to loading conditions and the geometry of the facet joint^32^. In addition, it has been documented that facet joint force is largely dependent on its orientation^22^. In the process of building a degenerated model, the relative positions of the two pieces of facet cartilage in the posterior element of the degenerated segment change. Therefore, abnormal facet contact forces can exist in the degenerated segment. Due to the reasons above, no apparent consistency was observed in the force at the facet joints in the degenerated segment as the degree of disc degeneration increased. In the degenerated segment, the load at the facet joint during lateral bending was considerably smaller than during axial rotation and extension. A similar phenomenon was found by Rohlmann *et al*.^22^. Such a phenomenon may be caused by the physiological anatomical structure of the lumbar facet joint^32^. However, the load in the facet joint of the segment adjacent to the L_4_–L_5_ degenerated segment increased with increasing degenerative pathology. Such a trend has been confirmed in previous studies of lumbar degeneration^29^. In addition, investigations have reported that excessive facet joint forces may result in painful articular facets^55^, ^56^. From this perspective, an abnormal increase in facet joint load in segments adjacent to L_4_–L_5_ is likely to be a cause of back pain. Overall, degenerated discs also affect posterior facet joints and cause abnormal forces. Therefore, in future finite element analyses of the spine, we must pay attention to the actual changes in the facet joint to better simulate the result of disc degeneration.

Degeneration of the lumbar disc causes an increase in stiffness in the degenerated segment, although the overall ROM of the spine is not much affected, inevitably resulting in a compensatory reduction in the stiffness of other segments, thereby increasing the ROM of other segments, with increased stress in the intervertebral disc and facet joint loading. Larger mechanical force may cause unrecoverable micro‐injury of the disc and facet joints, possibly accelerating the degeneration of adjacent segments over time^57^, ^58^. There will also be changes similar to lumbar disc degeneration after lumbar interbody fusion; that is, accelerated degeneration of adjacent segments. Previous studies have also shown that adjacent segmental degeneration (ASD) is widespread as a complication of lumbar fusion^59^, ^60^. For example, Tang *et al*. explored the relationship between ALIF and ASD and concluded that ALIF resulted in more adverse effects than disc degeneration on the biomechanical behavior of adjacent upper segments that may exacerbate adjacent upper segmental degeneration^27^. A number of researchers also think that the natural progression of degeneration results in adjacent segment degeneration, not lumbar fusion^59^. To summarize, for both lumbar disc degeneration and fusion, there is the risk of accelerating the degeneration of adjacent segments.

Degeneration of passive elements of the lumbar spine can cause abnormal stress^46^. Abnormal increases in stress at a degenerated segment and adjacent segments may be the cause of pain experienced by patients, but it remains unclear which is the dominant factor that causes pain. To our knowledge, degenerative changes in the ROM of the lumbar spine are the direct cause of abnormal stress. Therefore, in clinical treatments, excessive movement of the lumbar spine should be avoided when the lumbar spine of a patient becomes degenerated; otherwise it may lead to pain. Even if the pain is improved by lumbar fusion surgery, the spine should not be exposed to excessive motion. To some extent, lumbar disc degeneration and fusion result in the same biomechanical changes. It may be difficult for the patient's lumbar spine to return to the previous healthy ROM while avoiding pain. Therefore, understanding biomechanical changes in disc degeneration remains critical for further analysis of the mechanisms of lumbar spine disorders and improvements in clinical treatment.

There are several reasons for choosing a reduction in disc height, the formation of anterior osteophytes, endplate sclerosis, and a reduction in nucleus pulposus volume to simulate disc degeneration. First, there is a correlation between disc height loss, anterior osteophyte formation, and endplate sclerosis that has been demonstrated statistically^24^, ^45^. A previous radiographic study demonstrated that the endplate contour flattened with progressing disc degeneration^61^. However, endplate curvature in our lumbar finite element model was very small, so the endplate shape was not changed but endplate sclerosis was simulated by changing the material properties (increase in hardness). Second, a reduction in nucleus pulposus volume is also an important feature of lumbar disc degeneration^10^, ^13^. In the present study, we simulated the reduction in nucleus pulposus volume using a combination of decreased disc height and the cross‐sectional area of the nucleus pulposus. In addition, we modified the material properties of normal disc tissue to more accurately simulate the conditions of disc degeneration. Such morphological changes and variations in material properties are the principal features of intervertebral disc degeneration^12^, ^13^, ^22^, ^23^, ^24^, ^27^, ^28^, ^29^. Therefore, our degenerative model represents a certain proportion of cases of clinical degeneration cases and provides some guidance for clinical treatment.

The causes of disc degeneration are not fully understood. Patient complaints are varied. First, it may start with fracture (or other trauma) of the cartilage endplate, with abnormal load and calcification accelerating cartilage endplate damage, leading to more severe degeneration^62^. Second, loss of proteoglycans and a reduction in hydration of the nucleus pulposus can cause stress inside the disc to increase and, therefore, decrease the disc's subsequent capability to withstand physiological loads^63^. It is worth noting that cartilage endplate–vertebral endplate failure is definitely among the most important triggers for disc degeneration, because nutrition can be interrupted and cell viability of the intervertebral disc damaged^64^. In addition, a number of researchers suggest that if damage to the vertebral endplate is small, the flow of nutrients can be maintained. However, the quantity of nutrients may not be sufficient to maintain integrity of the disc^65^, ^66^. It can be seen from the above that the endplate affects disc degeneration by regulating the transport of nutrients. Therefore, it is necessary to construct a porous‐osmotic‐hyperelastic disc based on nutrient transport in future models.

The structure of the human lumbar spine is very complicated, resulting in a number of simplifications and limitations in our finite element analysis. First, disc degeneration is usually accompanied by degeneration of the posterior facet joint. In this FE study, we did not observe changes in facet joint geometry in the degenerated lumbar segments in experimental studies. Therefore, the posterior facet joint of degenerated segments is not accurately simulated. Second, the lumbar spine FE model in the present study was developed from geometric information from a single subject, while morphological differences between individuals may result in differences in motion patterns and locations of stress. Third, the lack of lumbar muscle is a common shortcoming in all current lumbar finite element models, although muscle plays an important role in regulating lumbar motion and maintaining stability^67^. Fourth, due to the difficulty in simulating the true geometry of the ligaments, they are simplified to one‐dimensional nonlinear spring elements. Despite these simplifications and limitations, the FE model of the lumbar spine developed here exhibited good consistency with published experimental data and will be useful in investigating the effects of disc degeneration on the lower lumbar spine. In addition, as lumbar disc degeneration FE models created by different researchers have different methods of modeling, loading and boundary conditions, it is difficult to compare quantitatively with the data from these models.

### *Conclusion*

In conclusion, the present study analyzed the biomechanical effects of L_4_–L_5_ segmental disc degeneration on the degenerated segment and healthy adjacent segments. We found that degenerated intervertebral discs led to changes in the motion and loading patterns of degenerated segments and adjacent normal segments. The abnormal loading and motion in the degenerated segment and its adjacent healthy segments risked accelerating degeneration in the adjacent segments. In addition, disc degeneration is usually accompanied by degeneration of the posterior facet joints, so accurate simulation of degenerated facet joints is essential for predicting changes in facet joint forces after disc degeneration. In the future, it may be necessary to determine the actual changes in the facet joints after lumbar disc degeneration through *in vitro* experimental studies.
